# Combining segmentation and attention: a new foveal attention model

**DOI:** 10.3389/fncom.2014.00096

**Published:** 2014-08-14

**Authors:** Rebeca Marfil, Antonio J. Palomino, Antonio Bandera

**Affiliations:** ISIS Group, Department of Electronic Technology, University of MálagaMálaga, Spain

**Keywords:** artificial attention, foveal images, foveal segmentation, saliency computation, irregular pyramids

## Abstract

Artificial vision systems cannot process all the information that they receive from the world in real time because it is highly expensive and inefficient in terms of computational cost. Inspired by biological perception systems, artificial attention models pursuit to select only the relevant part of the scene. On human vision, it is also well established that these units of attention are not merely spatial but closely related to perceptual objects (proto-objects). This implies a strong bidirectional relationship between segmentation and attention processes. While the segmentation process is the responsible to extract the proto-objects from the scene, attention can guide segmentation, arising the concept of foveal attention. When the focus of attention is deployed from one visual unit to another, the rest of the scene is perceived but at a lower resolution that the focused object. The result is a multi-resolution visual perception in which the fovea, a dimple on the central retina, provides the highest resolution vision. In this paper, a bottom-up foveal attention model is presented. In this model the input image is a foveal image represented using a Cartesian Foveal Geometry (CFG), which encodes the field of view of the sensor as a fovea (placed in the focus of attention) surrounded by a set of concentric rings with decreasing resolution. Then multi-resolution perceptual segmentation is performed by building a foveal polygon using the Bounded Irregular Pyramid (BIP). Bottom-up attention is enclosed in the same structure, allowing to set the fovea over the most salient image proto-object. Saliency is computed as a linear combination of multiple low level features such as color and intensity contrast, symmetry, orientation and roundness. Obtained results from natural images show that the performance of the combination of hierarchical foveal segmentation and saliency estimation is good in terms of accuracy and speed.

## 1. Introduction

Human vision system presents an interesting set of features of adaptability and robustness that allows it to analyse and process the visual information of a complex scene in a very efficient manner. Research in Psychology and Physiology demonstrates that the efficiency of natural vision has foundations in visual attention, which is a process that filters out irrelevant information and limits processing to salient items (Duncan, 1984). It has been demonstrated by psychophysics studies that, when a human observes a scene, she does not do so as a whole, but rather will make a series of visual fixations at salient locations in the scene using eye saccade movements (Martinez-Conde et al., 2004). These voluntary movements have the main purpose of capturing salient locations using the central region of the retina (fovea), which is the place where the human retina has a high concentration of cones and the image is captured with fine resolution. Psychophysics studies suggest other important role of fixations in how humans perceive a scene (Martinez-Conde et al., 2004). Experiments show that subjects are not able to detect scene changes when they occur at a location away from the fixation, unless they modify the gist of the scene. Because the scene is captured with less resolution in the periphery than in the fovea. In contrast, the changes are detected quickly when they occur in the fixation area or close to it. Then, it is clear that there is a relationship between visual fixation and attention in the human vision system. Attention allows to select salient locations that using a visual fixation are centered in the fovea to be acquired with fine resolution, while the rest of the scene is captured with less resolution. This multi-resolution encoding allows the human visual system to perceive a large field of view, bounding the data flow coming from the retina.

**Figure 1:**
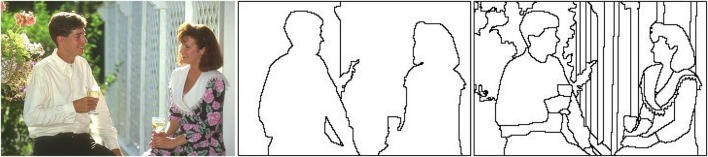
**(Left)** Test image #157055 from the Berkeley Segmentation Data Set (BSDS300) (Martin et al., 2001), **(Middle)** segmentation by user #1109 (8 segments), and **(Right)** segmentation by user #1123 (61 segments).

In the Computer Vision community, the non-uniform encoding of images has been emulated through methods such as the Reciprocal Wedge Transform (RWT), or the log-polar or Cartesian Foveal Geometries (CFG) (Traver and Bernardino, 2010). Also the selection of salient regions from an image has been widely studied, appearing different artificial attention models (Frintrop et al., 2010). However, the combination of attention and foveal image representation has been very little studied. This combination implies a close bidirectional relationship between foveal image segmentation and attention. This relationship comes from the fact that the location of human fixation is closely related to perceptual objects or proto-objects instead of disembodied spatial locations of the image (Rensink, 2000). Proto-objects can be defined as units of visual information that can be bounded into a coherent and stable object and they can be extracted using a perceptual segmentation algorithm. So, it seems logical to place the fovea in the location of the most salient proto-object in each moment. The saliency of each proto-object is obtained using an artificial attention model. Therefore the relationship between foveal segmentation and attention in one direction is clear: foveal segmentation provides the proto-objects to attention. But also the reverse relationship is very important. Segmentation essentially refers to a process that divides up a scene into non-overlapping, compact regions. Each region encloses a set of pixels that are bound together on the basis of some similarity or dissimilarity measure. A large variety of approaches for image segmentation has been proposed by the Computer Vision community in the last decades. And simultaneously, this community has been asked for a definition of what a correct segmentation is. As several authors have argued, the conclusion about this problem definition is that it is not well posed (Lin et al., 2007; Singaraju et al., 2008; Mishra et al., 2012). For example, if we see the original image and the segmentations provided by two human subjects in Figure 1, a major question arises: which is the correct segmentation? The answer to this question depends on what object we want to segment in the image: the two people (Figure 1 middle) or certain image details such as faces or hands (Figure 1 right). As Mishra et al. (2012) pointed out, the answer to this question depends on another question: what is the object of interest on the scene? Attention can be used to provide segmentation with the object of interest, fitting the correct input parameters and making segmentation well-defined (Jung and Kim, 2012; Mishra et al., 2012). These methods make use of the influence of attention in segmentation, but they do not take into account the reverse relation: how segmentation can influence attention.

In this paper, we propose a foveal attention mechanism which illustrates the bidirectional relation among attention and foveal segmentation. It uses a hierarchical image encoding where foveal segmentation and bottom-up attention processes can be simultaneously performed. As other approaches, this structure resembles the one of the human retina: it will only capture a small region of the scene in high resolution (fovea), while the rest of the scene will be captured in lower resolution on the periphery. Specifically, we use an adaptive CFG where the fovea can be located in any place of the scene and its size can be dynamically modified. The structure of the CFG is very suitable for hierarchical processing, allowing to encode the multi-resolution image within a *foveal polygon*. The foveal polygon represents the image at different resolution levels and is built using the irregular decimation process of the Bounded Irregular Pyramid (BIP) (Marfil et al., 2007) applied to perceptual segmentation. The saliency of each proto-object is computed following the *Feature Integration Theory* (Treisman and Gelade, 1980) as a linear combination of a set of low level features which clearly influences attention. While the computation of the low level features is independent of the task, being a pure bottom-up process, the linear combination of features is computed as a weighted summation where the weights can be set depending on the task in a top-down way. This attention mechanism is able to manage dynamic scenarios by adding an Inhibition of Return (IOR) mechanism which keeps permanently updated the position of each already attended proto-object and avoids revisiting an already attended one.

### 1.1. Related work

According to the taxonomy of computational models of visual attention proposed by Tsotsos and Rothenstein (2011), the method proposed in this paper can be considered as a saliency-based one. From the psychological point of view, the development of saliency-based computational models of visual attention is mainly based on the so-called *early-selection* theories. These theories postulate that the selection of a relevant region precedes pattern recognition. Therefore, attention is drawn by simple features (such as color, location, shape or size) and attended entities do not have full perceptive meaning, i.e., they could not correspond to real objects. Two complementary biological theories or descriptive models are the most influential ones regarding saliency-based computational models of visual attention: Treisman's *Feature Integration Theory* (FIT) (Treisman and Gelade, 1980) and Wolfe's *Guided Search* (Wolfe et al., 1989; Wolfe, 1994). FIT suggests that the human vision system detects separable features in parallel in an early step of the attention process. According to this model, methods compute image features in a number of parallel channels in a *pre-attentive* task-independent stage. Then, the extracted features are integrated through a *bottom-up* process into a single saliency map which codes the relevance of each image entity. The first saliency-based computational models mainly followed these guidelines. For example, the models proposed by Itti et al. (1998) or Koch and Ullman (1985) compute the saliency of each pixel based on a set of basic features. They were pure bottom-up, static models. Several years later, Wolfe proposed that a *top-down* component in attention can increase the speed of the process giving more relevance to those parts of the image corresponding to the current task. These two approaches are not mutually exclusive and, nowadays, several efforts in computational attention are being conducted to develop models which combine a bottom-up processing stage with a top-down selection process. Thus, Navalpakkam and Itti (2005) modified Itti's original model in order to add a multi-scale object representation in a long-term memory. The multi-scale object's features stored in this memory determine the relevance of the scene features depending on the current executed task, implementing, therefore, a top-down behavior. As an alternative to *space-based* models, where attention deploys on an unstructured region of the scene rather than on an object, *object-based* models of visual attention provide a more efficient visual search. These models are based on the assumption that the boundaries of segmented objects, and not just spatial position, determine what is selected and how attention is drawn (Scholl, 2001). Therefore, these models reflect the fact that perception abilities must be optimized to interact with objects and not just with disembodied spatial locations. Orabona et al. (2007) propose a model of visual attention based on the concept of *proto-objects* (Rensink, 2000) as units of visual information that can be bound into a coherent and stable object. They compute these proto-objects by employing the watershed transform to segment the input image using edge and color features in a pre-attentive stage. The saliency of each proto-object is computed taking into account top-down information about the object to perform a task-driven search. Yu et al. (2010) propose a model of attention that segments the scene into proto-objects in a bottom-up strategy based on Gestalt theories. After that, in a top-down way, the saliency of the proto-objects is computed taking into account the current task to accomplish by using models of objects which are relevant to this task. These models are stored in a long-term memory. These proto-object based models compute in a firs step the set of proto-objects from the scene and then they compute their saliency. There exist other type of methods that first compute the saliency map from the scene and then, the most salient proto-object is computed from the saliency map (Walther and Koch, 2006).

Attention theories introduce another important concept: the *Inhibition of Return* (IOR) (Posner et al., 1985). Human visual psychophysics studies have demonstrated that a local inhibition is activated in the saliency map to avoid attention being directed immediately to a previously attended region. In the context of computational models of visual attention, this IOR has been usually implemented using a 2D inhibition map that contains suppression factors for one or more focuses of attention that were recently attended (Itti et al., 1998; Frintrop, 2006). However, this 2D inhibition map is not able to handle the situations where inhibited objects are in motion or when the vision system itself is in motion. In this situation, establishing a correspondence between regions of the previous frame with those of the successive frame becomes a significant issue. In order to allow that the inhibition can track an object while it changes its location, the model proposed by Backer et al. (2001) relates the inhibitions to features of activity clusters. However, the scope of dynamic inhibition becomes very limited as it is related to activity clusters rather than objects themselves (Aziz and Mertsching, 2007). Thus, it is a better option to attach the inhibition to moving objects (Tipper, 1991). Aziz and Mertsching (2007) utilizes a queue of inhibited region features to maintain object inhibition in dynamic scenes.

Finally, Psychophysics studies also refer to how many elements can be attended at the same time. Bundesen establishes in his *Theory of Visual Attention* (Bundesen et al., 2011) that there exists a short-term memory where recently attended elements are stored. This memory has a fixed capacity usually reduced up to 3 or 5 elements.

All the attention models presented in this section have focused in different aspects such as e.g., the identification of features which influence attention, the combination of these features to generate the saliency map or how an specific task drives attention. But they neglect the foveal nature of the human attention system. The methods following a multi-resolution strategy usually employ two images of different resolution (Meger et al., 2008): A low-resolution image for computing the saliency map of the scene and a high resolution one for studying in detail the most salient region. Foveation has been typically proposed as an efficient way for image encoding (Geisler and Perry, 1998; Guo and Zhang, 2010). Built over the foveal encoding by Geisler and Perry (1998), the Gaze Attentive Fixation Finding Framework (GAFFE) (Rajashekar et al., 2008) employs four low-level local image saliency features (luminance, contrast, and bandpass outputs of both luminance and contrast) to build saliency maps and predict gaze fixations. It works on a sequential process in which the stimulus is foveated at the current fixation point and saliency features are obtained from circular patches from this foveated image to predict the next fixation point. This strategy has been recently evaluated by Gide and Karam (2012), replacing these saliency features with features from other models such as AIM (Attentive Information Maximization) (Bruce and Tsotsos, 2009) or SUN (Saliency Using Natural Image Statistics) (Zhang et al., 2008). Evaluated under a quality assessment task for different types of distortions (Gaussian blur, white noise and JPEG compression), Gide and Karam (2012) showed that the performance of all saliency models significantly improved with foveation over all distortion types. It should be noted that Rajashekar et al. (2008) and Gide and Karam (2012) do not obtain the fixation points from a saliency model, but from features extracted of the foveated images. Following a different strategy, Advani et al. (2013) propose to encode the image as a three level Gaussian pyramid. The higher level represents the whole field-of-view at a lower resolution, meanwhile the lower one only encodes the 50% of the field-of-view at the resolution of the original image. The AIM model is run at these three levels, which returns corresponding information maps. These maps represent the salient regions at different resolutions and are fused within an unique saliency map using weighted summation.

### 1.2. Overview of the proposed attention model

In this paper, a bottom-up foveal attention model is presented. The input of this model is a foveal image represented in an adaptive CFG where the focus of attention, or Region of Interest (ROI), is located at the fovea which is surrounded by a set of concentric rings with decreasing resolution. In this model the attention is deployed to proto-objects instead of disembodied spatial locations. These proto-objects are defined as the blobs of uniform color and disparity of the image which are bounded by the edges obtained using a Canny detector. They are extracted using a perceptual segmentation algorithm which is conducted using an extension of the BIP (Marfil et al., 2007). The saliency of each proto-object is computed in a bottom-up framework in order to obtain the ROI for the next frame. This saliency value is the combination of a set of low level features that according to psychological studies clearly influences saliency computation (Treisman and Souther, 1985; Wolfe et al., 1992). Specifically, it is computed in terms of the following features: color contrast, intensity contrast, proximity, symmetry, roundness, orientation and similarity to skin color. To have an homogenized calculus, all features values are normalized in the range [0 … 255].

Hence, contrary to all previous approaches to foveal attention, our approach merges within the same hierarchical framework the segmentation and saliency estimation processes. The levels of the hierarchy are not obtained by blurring and downsampling the content on the level below and adding additional information to increase the field-of-view. In our approach, each level of the hierarchy is able to provide a segmentation of the encoded field-of-view. Then, the highest level of the hierarchy, that encodes the full field-of-view, provides a segmentation *S*^*t*^ where the fovea details are present but those at the peripheral regions are not. This segmentation *S*^*t*^ depends on the fovea location provided by the attention process at *t* − 1 and drives the next location of the fovea. Once the saliency of each proto-object is computed, the ROI at *t* + 1 is extracted as the location of the most salient proto-object in the current frame. In order to compute this ROI and to avoid revisiting or ignoring proto-objects, it is necessary to implement an Inhibition of Return mechanism (IOR). This IOR is very important in the case of dynamic environments where there are moving objects. It is typically implemented using a 2D inhibition map which contains suppression factors for one or more recently attended focuses of attention. This approach is valid to manage static scenarios, but it is not able to handle dynamic environments where inhibited proto-objects or the vision system itself are in motion. In the proposed system, a tracker module keeps permanently updated the position of recently attended proto-objects or focuses of attention. The features and location of these already attended proto-objects are stored in a Working Memory. Thereby, it is avoided to attend an already selected proto-object even if the proto-object changes its location in the image. Specifically, the tracker is based on the Comaniciu mean-shift approach (Comaniciu et al., 2003), a method which allows to track non-uniform color regions in an image.

**Figure 2:**
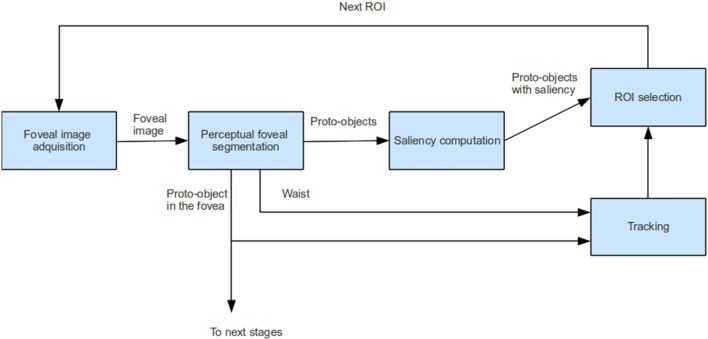
**Overview of the proposed foveal attention model**.

**Figure 3:**
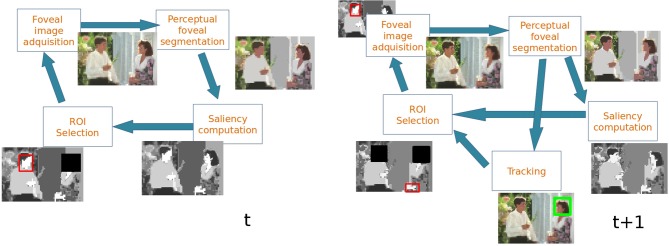
**Example of the operation of the system in two consecutive frames**.

Figure 2 shows the main stages involved in the proposed attentional model and Figure 3 shows an example. First, a foveal image is captured with the fovea located in the Region of Interest (ROI) computed in the previous frame. In frame *t* of Figure 3 the fovea is located in the woman's face, in *t* + 1 the fovea is located in the man's face. It must be noted that in the first frame the fovea is located at the image center. After that, the foveal image is segmented by building the Foveal Polygon using the BIP. In this stage the set of proto-objects is extracted from the foveal image and the fovea could be processed by further attentional stages (that are out of the scope of this paper). Then, saliency of each obtained proto-object is computed. These saliency values are used to compute the ROI of the next frame taking into account the output of the tracking module. This tracker computes the locations of the previously attended proto-objects in the current frame. These locations and the location of the current ROI are inhibited in order to extract the new ROI (black squares in Figure 3).

### 1.3. Contributions

The main contributions of this work are:

- The use of foveal images as inputs of the attentional mechanism.
- The hierarchical representation of the foveal image that allows to simultaneously built the foveal polygon and perceptually segment the input image extracting the proto-objects.
- The combination of foveal segmentation and attention: the attention process allows to select the next position of the fovea and segmentation allows to extract the units of attention.

### 1.4. Organization of the paper

After providing a brief overview of the proposed approach in this Section 1, the rest of the paper is organized as follows: Sections 2, 3 provide a more detailed description of the two main processes (perceptual foveal segmentation and bottom-up attention) tied within our framework. Section 2 introduces the Cartesian Foveal Geometries and the concept of the Foveal Polygon. Then, it describes the data structure and decimation strategy that define the foveal Bounded Irregular Pyramid (foveal BIP). Section 3 describes how the saliency is computed and the ROI is chosen, including a description of our implementation of the IOR. Section 4 evaluates the performance of the foveal attention system. Three kinds of tests have been conducted: a comparison of the uniform and foveated models of attention, an evaluation of the ability of our approach for actively driving an image exploration process, and a quantitative evaluation of the attention and fixation prediction models.

## 2. Perceptual foveal segmentation

In this paper, we propose an artificial attentional system which uses a hierarchical image encoding where segmentation and bottom-up attention processes are simultaneously performed. This image encoding resembles the one of the human retina by using a foveal representation: only a small region of the scene is captured with high resolution (fovea), while the rest of the scene is captured in lower resolution on the periphery. Specifically, an adaptive Cartesian Foveal Geometry is used to capture the input image which is hierarchically encoded by means of a Perceptual Segmentation approach. It allows to extract the proto-objects from the visual scene and it is conducted using the Bounded Irregular Pyramid (BIP) (Marfil et al., 2007).

### 2.1. Cartesian Foveal Geometries (CFG) and foveal polygons

**Figure 4:**
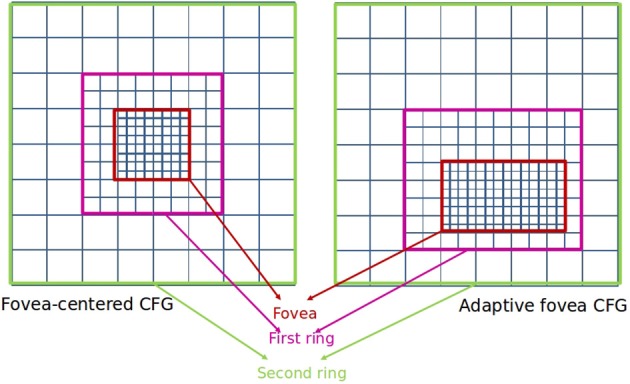
**Cartesian Foveal Geometries (CFG)**.

Cartesian Foveal geometries (CFG) encode the field of view of the sensor as a fovea surrounded by a set of concentric rings with decreasing resolution (Arrebola et al., 1997). In the majority of the Cartesian proposals, this fovea is centered on the geometry and the rings present the same parameters. Thus, the geometry is characterized by the number of rings surrounding the fovea (*m*) and the number of subrings of resolution cells (*rexels*) found in the directions of the Cartesian axes within any of the rings. Figure 4 shows an example of a fovea-centered CFG.

**Figure 5:**
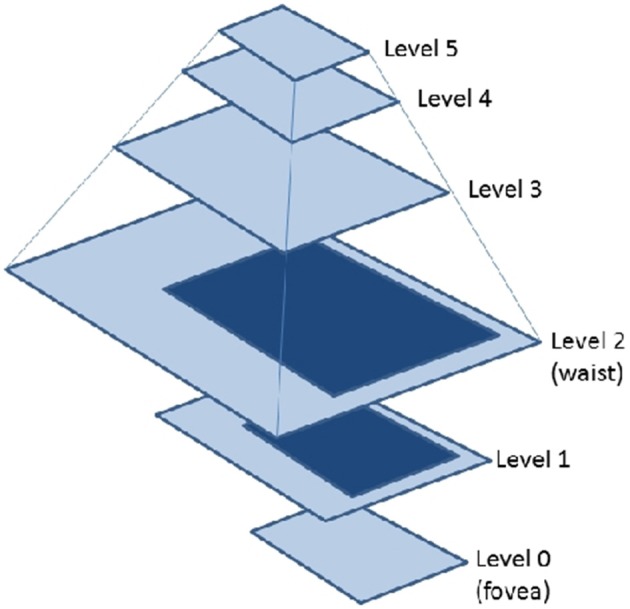
**Foveal Polygon associated to an adaptive CFG with two rings**.

Among other advantages, there are CFGs that are able to provide a shiftable fovea of adaptive size (Arrebola et al., 1997) (adaptive CFGs). Vision systems which use the fovea-centered CFG require to place the region of interest in the center of the image. That is usually achieved by moving the cameras. A shiftable fovea can be very useful to avoid these camera movements. Furthermore, the adaptation of the fovea to the size of the region of interest can help to optimize the consumption of computational resources. Figure 4 shows the rectangular structure of an adaptive fovea. The geometry is now characterized by the subdivision factors at each side of the fovea. It should be noted that the foveal geometry is not adequate for processing planar images. On the contrary, the aim is to use it for hierarchical processing. Thus, a hierarchical representation of the foveal image (the foveal polygon) is built like Figure 5 shows. This foveal polygon has a first set of levels of abstraction built from the fovea to the waist (the first level where the complete field of view is encoded). In the figure, levels 1 and 2 on this hierarchy are built by decimating the information from the level below and adding the data from the corresponding ring of the multi-resolution image. Over the waist, there are a second set of levels. All these levels encode the whole field of view and are built by decimating the level below.

Typically, the decimation process inside the CFGs have been conducted using regular approximations (Arrebola et al., 1997). Then, all levels of the foveal polygon can be encoded as images. The problems of regular decimation processes were early reported (Antonisse, 1982; Bister et al., 1990), but here, these processes were justified due to the simplicity for processing (Traver and Bernardino, 2010).

In this work, we propose to build the foveal polygon using the irregular decimation process provided by the Bounded Irregular Pyramid (BIP) (Marfil et al., 2007).

### 2.2. Perceptual foveal segmentation using BIP

The BIP is an irregular pyramid which is defined by a data structure and an irregular decimation process. This irregular decimation is applied to build the foveal polygon by segmenting the foveal input image using a perceptual segmentation approach which allows to extract the proto-objects from the visual scene.

#### 2.2.1. Data structure of the BIP

The data structure of the BIP is a mixture of regular and irregular data structures: a 2 × 2/4 “incomplete” regular structure and a simple graph. The regular structure of the BIP is said to be incomplete because, although the whole storage structure is built, only the homogeneous regular nodes (see subsection 2.2.2) are set in it. Therefore, the neighborhood relationships of these nodes can be easily computed. The mixture of both regular and irregular structures generates an irregular configuration which is described as a graph hierarchy. In this hierarchy, there are two types of nodes: nodes belonging to the 2 × 2/4 structure, named *regular nodes* and *irregular nodes* or nodes belonging to the irregular structure. Therefore, a level *l* of the hierarchy can be expressed as a graph *G*_*l*_ = (*N*_*l*_, *E*_*l*_), where *N*_*l*_ stands for the set of regular and irregular nodes and *E*_*l*_ for the set of arcs between nodes (intra-level arcs). Each node *n*_*i*_ ∈ *N*_*l*_ is linked with a set of nodes {*n*_*k*_} of *N*_*l* − 1_ using inter-level arcs, being {*n*_*k*_} the reduction window of *n*_*i*_. A node *n*_*i*_ ∈ *N*_*l*_ is neighbor of other node *n*_*j*_ ∈ *N*_*l*_ if their reduction windows *w*_*n*_*i*__ and *w*_*n*_*j*__ are connected. Two reduction windows are connected if there are at least two nodes at level *l*-1, *n*_*p*_ ∈ *w*_*n*_*i*__ and *n*_*q*_ ∈ *w*_*n*_*j*__, which are neighbors.

#### 2.2.2. Decimation process of the foveal BIP

Two nodes *x* and *y* which are neighbors at level *l* are connected by an intra-level arc (*x*, *y*) ∈ *E*_*l*_. Let ε^*xy*^_*l*_ be equal to 1 if (*x*, *y*) ∈ *E*_*l*_ and equal to 0 otherwise. Then, the neighborhood of the node *x* (ξ_**x**_) can be defined as ξ_**x**_ = {**y** ∈ *N*_*l*_ : ε^**xy**^_*l*_}. It can be noted that a given node **x** is not a member of its neighborhood, which can be composed by regular and irregular nodes. Each node **x** has associated a *v*_**x**_ value. Besides, each regular node has associated a boolean value *h*_**x**_: the homogeneity (Marfil et al., 2007). At the base level of the hierarchy *G*_0_, the fovea, all nodes are regular, and they have *h*_**x**_ equal to 1 (they are homogeneous). Only regular nodes which have *h*_**x**_ equal to 1 are considered to be part of the regular structure. Regular nodes with an homogeneity value equal to 0 are not considered for further processing. The proposed decimation process transforms the graph *G*_*l*_ in *G*_*l* + 1_ using the pairwise comparison of neighbor nodes. Then, a pairwise comparison function, *g*(*v*_**x_1_**_, *v*_**x_2_**_) is defined. This function is true if the *v*_**x**__1_ and *v*_**x**__2_ values associated to the **x**_1_ and **x**_2_ nodes are similar according to some criteria and false otherwise. When *G*_*l* + 1_ is obtained from *G*_*l*_, being *l* < waist, this graph is completed with the regular nodes associated to the ring *l* + 1. This process will require to compute the neighborhood relationships among the regular nodes coming from the ring and the rest of nodes at *G*_*l* + 1_. Over the waist level, *G*_*l* + 1_ is built by decimating the level below *G*_*l*_.

The building process of the foveal BIP consists of the following steps:

1. Regular decimation process. The *h*_**x**_ value of a regular node **x** at level *l* + 1 is set to 1 if the four regular nodes immediately underneath {**y**_*i*_} are similar according to some criteria and their *h*_{**y**__*i*}_ values are equal to 1. That is, *h*_**x**_ is set to 1 if
  (1) $$\left\{\underset{\forall y_{j},y_{k}\in \{y_{i}\}}{\cap }g\left(v_{y_{j}},v_{y_{k}}\right)\}\cap \{\underset{y_{j}\in \{y_{i}\}}{\cap }h_{y_{j}}\right\}$$
  Besides, at this step, inter-level arcs among homogeneous regular nodes at levels *l* and *l* + 1 are established. If **x** is an homogeneous regular node at level *l* + 1 (*h*_**x**_ = = 1), then the set of four nodes immediately underneath {**y_*i*_**} are linked to **x** and the *v*_*x*_ value is computed.
2. Irregular decimation process. Each irregular or regular node **x** ∈ *N*_*l*_ without parent at level *l* + 1 chooses the closest neighbor **y** according to the *v*_**x**_ value. Besides, this node **y** must be similar to **x**. That is, the node **y** must satisfy
  (2) $$\left\{||v_{x}-v_{y}||=\min\left(||v_{x}-v_{z}||:z\in \xi_{x}\right)\}\cap \{g\left(v_{x},v_{y}\right)\right\}$$
  If this condition is not satisfied by any node, then a new node **x**′ is generated at level *l* + 1. This node will be the parent node of **x** and it will constitute a root node. Its *v*_*x*′_ value is computed. On the other hand, if **y** exists and it has a parent **z** at level *l* + 1, then **x** is also linked to **z**. If **y** exists but it does not have a parent at level *l* + 1, a new irregular node **z**′ is generated at level *l* + 1 and *v*_*z*′_ is computed. In this case, the nodes **x** and **y** are linked to **z**′.
  This process is sequentially performed and, when it finishes, each node of *G*_*l*_ is linked to its parent node in *G*_*l* + 1_. That is, a partition of *N*_*l*_ is defined. It must be noted that this process constitutes an implementation of the union-find strategy.
3. Definition of intra-level arcs. The set of edges *E*_*l* + 1_ is obtained by defining the neighborhood relationships between the nodes *N*_*l* + 1_. As aforementioned, two nodes at level *l* + 1 are neighbors if their reduction windows are connected at level *l*.
4. For *l* < waist
  - The set of nodes *N*_*l* + 1_ is completed with the rexels of the ring *l* + 1. These rexels are added as homogeneous regular nodes, *N*^ring^_*l* + 1_.
  - The intra-level arcs between nodes of *N*^ring^_*l* + 1_ and the rest of nodes of *N*_*l* + 1_ are computed as in step 3. Nodes of *N*^ring^_*l* + 1_ do not have a real reduction window at level *l*, they present a *virtual reduction window*. The virtual reduction window of a node **x** ∈ *N*^ring^_*l* + 1_ is computed by quadrupling this node at level *l*. Therefore, the reduction window of **x** is formed by the four nodes immediately underneath at level *l*.

**Figure 6:**
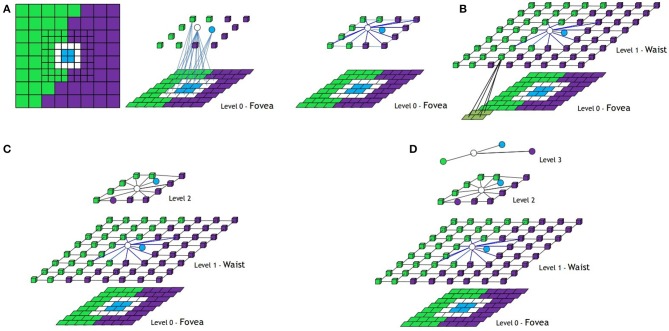
**Foveal image with one ring and how the structure of the Bounded Irregular Pyramid associated to it is built**. **(A)** Building the central part of Level 1 from the fovea (Level 0), **(B)** definition of the intra-level edges at this part and adding new nodes from Ring 1, **(C)** Building Level 2, and **(D)** Building Level 3. Regular nodes are drawn as 3d cubes and irregular ones as spheres (see text).

In Figure 6 the whole process to build the structure of the BIP associated to a foveal image with one ring is shown. Homogeneous regular nodes are represented by squares or cubes and irregular ones by spheres. In the first row, the process to build the first level is shown. From left to right: original image, nodes of the first level generated after the regular and irregular decimation processes (only some inter-level arcs are shown), structure of the first level after the definition of the intra-level arcs and final structure of the first level after adding the nodes of the ring (the virtual reduction window of one node of the ring is shown). In the second row of the figure, the rest of levels are shown.

#### 2.2.3. Perceptual segmentation

As the process to group image pixels into higher-level structures can be computationally complex, perceptual segmentation approaches typically combine a pre-segmentation step with a subsequent perceptual grouping step. The pre-segmentation step performs the low-level definition of segmentation. It groups pixels into homogeneous clusters. Thereby, pixels in input image are grouped into blobs of uniform color, replacing the pixel-based image representation. Besides, these regions preserve the image geometric structure because each significant feature contains at least one region. The perceptual grouping step conducts a domain-independent grouping which is mainly based on properties such as proximity, closure or continuity. Both steps are conducted using the aforementioned decimation process but employing different similarity criteria between nodes.

In order to compute the pre-segmentation stage, a basic color segmentation is applied. In this case, a distance based on the HSV color space is used. Two nodes *n*_*i*_ and *n*_*j*_ are similar (they share a similar color) if their HSV values are less or equal than a similarity threshold τ_color_:

(3) $$g(v_{n_{i}},v_{n_{j}})=(d(n_{i},n_{j}))\leq \tau_{\text{color}})$$

being *v*_**n**__*i*_ and *v*_**n**__*j*_ the HSV color of nodes *n*_*i*_ and *n*_*j*_ in cylindrical coordinates, and *d*(*n*_*i*_, *n*_*j*_) is the color distance between them.

(4) $$d(n_{i},n_{j})=\sqrt{d_{v}(n_{i},\;n_{j})+d_{c}(n_{i},\;n_{j})}$$

where

(5) $$d_{v}(n_{i},n_{j})=|V_{i}-V_{j}|$$

(6) $$d_{c}(n_{i},n_{j})=\sqrt{S_{i}+S_{j}+2\cdot S_{i}\cdot S_{j}\cdot \cos\theta }$$

with θ = |*H*_*i*_ − *H*_*j*_|.

In the perceptual grouping step, the roots of the pre-segmented blobs are considered the first level of a new segmentation process. In this case, two constraints are taken into account for an efficient grouping process: first, although all groupings are tested, only the best groupings are locally retained; and second, all the groupings must be spread on the image so no part of the image takes advantage. As segmentation criterion, a more complex distance is employed instead of a simple color threshold. This distance has three main components: the color contrast between blobs, the edges of the original image, obtained using a Canny detector, and the depth information of the image blobs in form of disparity. To avoid working at pixel resolution, which decreases the computational speed, a global contrast measurement is used instead of a local one. Then, the distance ϕ(*n*_*i*_, *n*_*j*_) between two nodes *n*_*i*_ and *n*_*j*_ is defined as:

(7) $$\phi (n_{i},n_{j})=\sqrt{\omega_{1}{\left[\frac{d(n_{i},n_{j})\cdot b_{i}}{\alpha \cdot C_{ij}+\beta (b_{ij}-c_{ij})}\right]}^{2}+\omega_{2}{\left[\delta (n_{i})-\delta (n_{j})\right]}^{2}}$$

where *d*(*n*_*i*_, *n*_*j*_) is the HSV color distance between *n*_*i*_ and *n*_*j*_, δ(*x*) is the mean disparity associated to the base image region represented by node *x*, *b*_*i*_ is the perimeter of *n*_*i*_, *b*_*ij*_ is the number of pixels in the common boundary between *n*_*i*_ and *n*_*j*_ and *c*_*ij*_ is the set of pixels in this common boundary which corresponds to pixels of the boundary obtained using the Canny detector. α and β are two constant values used to control the influence of the Canny edges in the grouping process. ω_1_ and ω_2_ are two constants which weight the terms associated with color and disparity. These parameters should be manually tuned depending on the application and the environment. Two nodes are similar if the distance ϕ(*n*_*i*_, *n*_*j*_) between them is equal or less than a threshold τ_percep_:

(8) $$g(v_{n_{i}},v_{n_{j}})=\left(\phi (n_{i},n_{j})\right)\leq \tau_{\text{percep}})$$

**Figure 7:**
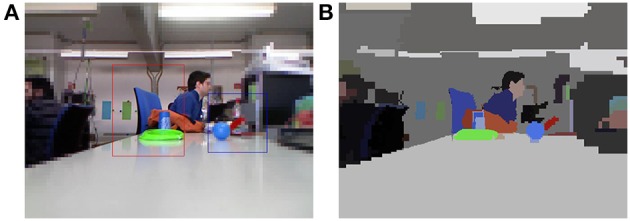
**(A)** Foveal image; **(B)** Perceptual segmentation associated to **(A)** (τ_color_ = 50, τ_percep_ = 100).

The grouping process is iterated until the number of nodes remains constant among two consecutive levels, because it is not possible to group together more nodes because they are not similar. After the perceptual grouping, the nodes of the BIP with no parents are the roots of the proto-objects. Figure 7 shows an example of the result of a perceptual segmentation.

## 3. Saliency computation and ROI selection

Once the scene is divided into proto-objects, the next step is the selection of the most relevant one. According to Treisman and Gelade (1980), this process is based on the computation of a set of low-level features. But, what features must be taken into consideration? What features really guide attention?

According to psychological studies, some features, such as color (Treisman and Souther, 1985), motion (McLeod et al., 1988) or orientation (Wolfe et al., 1992), clearly influence in saliency computation. These three features, plus size, are cataloged by Wolfe and Horowitz (2004) as the only undoubted attributes that can guide attention. Wolfe also offers in his work a complete list of features that might guide the deployment of attention, grouped by their likelihood to be an effective source of attentional guiding. He differentiates among the aforementioned undoubted attributes, probable attributes, possible attributes, doubtful cases and probable non-attributes.

Another important issue when selecting features to develop an artificial attention system is concerned with computational cost. Computing a large number of features provides a richer description about elements in the scene. However, the associated computing time could be unacceptable. Hence, it is necessary a trade-off between computational efficiency and the number and type of the selected features.

Following the previous guidelines, seven different features have been selected to compute saliency in the proposed system. From the undoubted attributes, orientation and color have been chosen. Because there is no background subtraction in the perceptual segmentation, larger proto-objects usually correspond to non-relevance parts of the image (e.g., walls, floor or empty tables). Therefore, size feature is not employed to avoid an erroneous highlighting of irrelevant elements. Motion is discarded due to computational cost restriction. Although intensity contrast is not considered an undoubted feature, it has also been included as a special case of color contrast (intensity deals with gray, black, and white elements). From the remainder of available possible attributes, those describing shape and location have been considered as more suitable for a complete description of the objects in scene. Location is calculated in terms of proximity to the visual sensor. Regarding the shape, two features are taken into account: symmetry, which allows to discriminate between symmetric and non-symmetric elements, and roundness, a measure about the closure and the contour of an object. Finally, in order to reach a social interaction with humans, it seems to be reasonable to include features able to pop out people from a scene. Although some works directly consider faces as a feature (Judd et al., 2009), experimental studies differ (Nothdurft, 1993; Suzuki and Cavanagh, 1995). Faces themselves do not guide attention but they can be separated into basic features that really achieve the guidance (Wolfe and Horowitz, 2004). In general, global properties are correlated with low-level features that explain search efficiency (Greene and Wolfe, 2011). Consequently, the proposed model uses similarity with skin color as an undoubted feature to guide attention to human faces in combination with other features as roundness.

**Figure 8:**
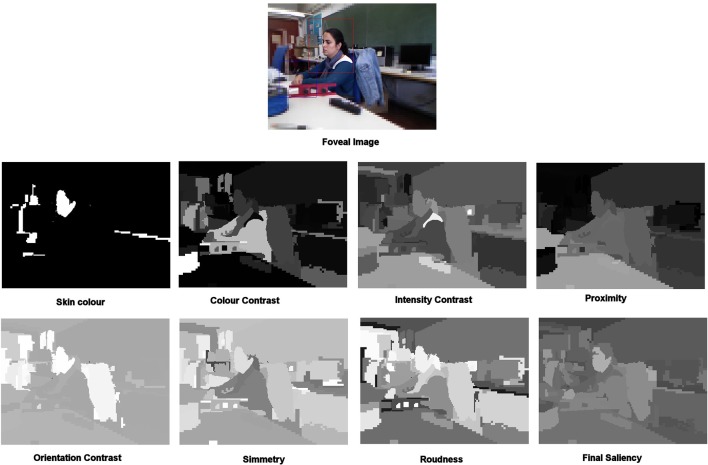
**Foveal image and its associated feature maps**.

To summarize, saliency is computed in terms of the following features: color contrast, intensity contrast, proximity, symmetry, roundness, orientation and similarity to skin color. All features values are normalized in the range [0 … 255] in order to have an homogenized calculus. As most of the artificial attention systems following Treisman's *Feature Integration Theory* (Treisman and Gelade, 1980), the total saliency of an element in an image is the result of a linear combination of its low-level features. Figure 8 shows an example of foveal image and its associated feature maps. These feature maps represent the value of the corresponding feature for each proto-object. The final saliency map is also shown.

**Figure 9:**
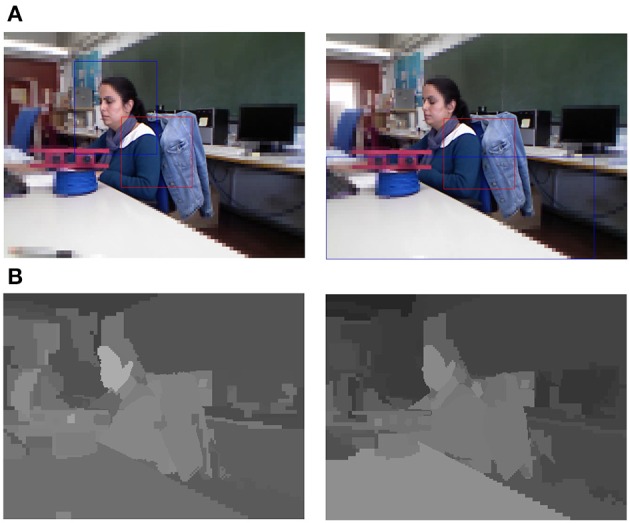
**(A)** First frames of two very similar sequences where the red box corresponds with the current fovea and the blue box corresponds with the next ROI; **(B)** Saliency maps obtained with all the weights set to 1/7 (left image) and with the weight corresponding to proximity equal to 0.5 and the rest to 0.5/6 (right image).

In the proposed attention system, the final saliency value, *sal*_*i*_, for each proto-object, 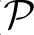
_*i*_, is obtained as a weighted sum of all the previously described features:

(9) $$sal_{i}=\vec{\lambda }\cdot \vec{f}$$

where $\vec{\lambda }$ is a set of weights, verifying $\sum_{i}\lambda_{i}=1$, and $\vec{f}$ is the feature vector formed by the different features computed as explained in the following subsections. As it was previously commented in the Introduction section the weights can be set depending on the task in a top-down way. For example, in Figure 9 two saliency maps obtained with a different set of weights are shown. While, in the left saliency map all the weights are set to the same value, in the right map the weight associated to the proximity feature is higher than the rest, and therefore, the proto-objects closer to the camera have a bigger saliency value than those who are far away. This variation in the saliency values causes a modification in the location of the next fovea (blue boxes in a). Therefore, the sequence of fixations of a scene can be modified by varying the values of the weights.

### 3.1. Color contrast and intensity contrast

These features measure how different a proto-object is with respect to its surrounding in terms of color and luminosity. The color contrast, (*ColCON*), of a specific proto-object, 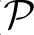
_*i*_, can be computed as the mean color gradient along its boundary to the neighbors in the segmentation hierarchy:

(10) $$ColCON_{i}=\frac{S_{i}}{b_{i}}\sum_{j\in N_{i}}b_{ij}\cdot d\left(<C_{i}>,<C_{j}>\right)$$

where *b*_*i*_ is the perimeter of 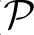
_*i*_, *N*_*i*_ is the set of proto-objects that are neighbors of 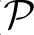
_*i*_, *b*_*ij*_ is the length of the perimeter of 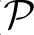
_*i*_ in contact with proto-object 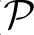
_*j*_, *d*[< *C*_*i*_ >, < *C*_*j*_ >] is the HSV color distance between the color mean values < *C* > of proto-objects 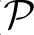
_*i*_ and 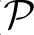
_*j*_ and *S*_*i*_ is the mean saturation value of proto-object 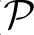
_*i*_.

Because of the use of *S*_*i*_ in the color contrast equation, white, black and gray proto-objects are suppressed. Thus, a feature about intensity contrast is also introduced. The intensity contrast, (*IntCON*), of a proto-object, 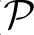
_*i*_, is computed as the mean luminosity gradient along its boundary to the neighbors:

(11) $$IntCON_{i}=\frac{1}{b_{i}}\sum_{j\in N_{i}}b_{ij}\cdot d\left(<I_{i}>,<I_{j}>\right)$$

being < *I*_*i*_ > the mean luminosity value of the proto-object 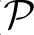
_*i*_.

### 3.2. Proximity

Another important parameter in order to characterize a proto-object is to determine its distance to the vision system. Nowadays, not only stereo pairs of cameras but also cheaper devices like Microsoft Kinect or ASUS Xtion provide accurate depth information of the captured image.

When using a sensor able to directly provide depth information (e.g., a RGBD camera or similar), the proximity, (*PROX*), of a proto-object, 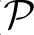
_*i*_, is directly obtained as the inverse of the mean of the depth values provided by the sensor in the area of the proto-object *depth*_*i*_:

(12) $$PROX_{i}=\frac{1}{depth_{i}}$$

In the case of using a stereo pair of cameras as depth sensor, the proximity can be obtained directly from disparity information.

### 3.3. Roundness

Roundness measurement reflects how similar to a circle a proto-object is. This feature provides information about convexity, closure and dispersion. Roundness is obtained employing a traditional technique based on image moments. Concretely, three different central moments are used:

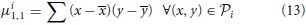

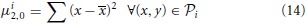

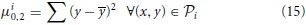







being (*x*, *y*) the center of the proto-object 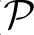
_*i*_.

From the combination of the equations above, it is possible to measure the difference between a region and a perfect circle. This measure is known as eccentricity and can be calculated as follows:

(16) $$ecc_{i}=\frac{{\left(\mu_{2,0}^{i}-\mu_{0,2}^{i}\right)}^{2}+{\left(2\mu_{1,1}^{i}\right)}^{2}}{{\left(\mu_{2,0}^{i}+\mu_{0,2}^{i}\right)}^{2}}$$

being the result in the range [0…1].

Finally, the roundness, (*ROUND*_*i*_), for a proto-object, 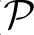
_*i*_, is obtained from the definition of eccentricity as:

(17) $$ROUND_{i}=1-ecc_{i}$$

### 3.4. Orientation

The orientation of a region in a image can also be obtained from central moments computed in (13–15):

(18) $$\varphi_{i}=\frac{1}{2}\arctan\left(\frac{2\mu_{1,1}^{i}}{\mu_{2,0}^{i}-\mu_{0,2}^{i}}\right)$$

But the orientation of a proto-object, by itself, does not provide any useful information about its relevance. Only when comparing its orientation with the orientation of the rest of proto-objects in the image, a feasible measure of relevance is obtained. Thus, in fact, it is more interesting to compute saliency in terms of contrast with the surrounding elements. The orientation contrast, (*OriCON*), of a proto-object, 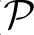
_*i*_, is obtained as:

(19) $$OriCON_{i}=\sum_{j\in N_{i}}\left|\varphi_{i}-\varphi_{j}\right|$$

where *N*_*i*_ is the set of proto-objects that are neighbors of 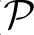
_*i*_.

Although pure orientation information is not employed to calculate relevance, it is saved as a descriptor of the proto-object for further use (for example, to compute symmetry).

### 3.5. Symmetry

To compute the symmetry of a proto-object, an approach similar to Aziz and Mertsching (2008) is followed. They propose a method to obtain symmetry using a scanning function ψ(*L*, *P*_*s*_) that counts the symmetric points around a point *P*_*s*_ along a line *L*. This procedure is repeated employing different lines of reference. For each line, the measure of symmetry is computed as:

(20) $$S^{\theta }=\sum_{s=1}^{l}\frac{\psi (L,P_{s})}{\alpha (R_{i})}$$

where *l* and θ are the length and the angle of the line of reference and α(*R*_*i*_) is the area of the region in order to normalize the result between 0 and 1.

Only an approximation of symmetry is needed in terms of attention systems. Thus, only 4 different angles for symmetry axes are considered: 0, 45, 90, and 135° respect to the orientation, φ_*i*_, of the image [obtained in (18)]. In Aziz and Mertsching (2008), the total measure of symmetry is computed as an average of the symmetry values in the different lines of reference. Nevertheless, such strategy can define a region with only one axis of symmetry as asymmetric, because non-symmetric axes cancel out the contribution of the symmetric one.

As relevance is given to symmetry independently of the axis of symmetry, the maximum symmetry, (*SYMM*), for a proto-object, 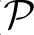
_*i*_, is computed as:

(21) $$SYMM=max_{\theta }(S^{\theta })$$

### 3.6. Skin color

The computation is based on the skin color chrominance model proposed by Terrillon and Akamatsu (1999). First, the image is transformed into the TSL color space. Then, the Mahalanobis distance between the color of the proto-object and the mean vector of the skin chrominance model is computed. If this distance is less than a threshold Θ_skin_, the skin color feature is marked with a value of 255. Otherwise, it is set to 0.

(22) $$SKN_{i}=\{\begin{array}{l} 255\text{ if }d_{M}\left(<C_{i}^{TSL}>,<C_{yellow}^{TSL}>\right)\leq \Theta_{skin}\\ 0\text{ otherwise } \end{array}$$

### 3.7. Inhibition of return and ROI selection

Once the saliency of each proto-object has been computed, the most salient one is selected as the next ROI where the fovea will be located in the next frame. In this process it is necessary to take into account that revisiting already attended proto-objects and ignoring not attended ones must be avoided. To do that an inhibition of return algorithm should be implemented.

Psychophysics studies about human visual attention have established that a local inhibition is activated in the saliency map when a region is already attended. This mechanism avoids directing focus of attention to a region immediately visited and it is normally called *inhibition of return (IOR)* (Posner et al., 1985). In order to handle dynamic environments, this IOR mechanism needs to establish a correspondence between regions among consecutive frames. In order to associate this inhibition to the computed proto-objects and not only to activity clusters as in Backer et al. (2001) or to object features as in Aziz and Mertsching (2007), an object-based inhibition of return applying image tracking is employed instead in the proposed work. To do that, recently attended proto-objects are stored in a Working Memory (WM). When the vision system moves, the proto-objects stored in the WM are kept tracked. In the next frame, a new set of proto-objects is obtained from the image and the positions of the previously stored ones are updated. Then, from the new set of proto-objects, those occupying the same region than the already attended ones are suppressed. Discarded proto-objects are not taken into account in the selection of the most salient one.

A tracker based on Dorin Comaniciu's *mean-shift* approach (Comaniciu et al., 2003) is employed to achieve the inhibition of return. Mean-shift algorithm is a non-parametric density estimator that optimizes a smooth similarity function to find the direction of movement of a target. A mean-shift based tracker is specially interesting because of its simplicity, efficiency, effectiveness, adaptability and robustness. Moreover, its low computational cost allows to track several objects in a scene maintaining a reasonable frame rate (real-time tracking of multiple objects). In the proposed system, the target model is represented by a 16-bin color histogram masked with an isotropic kernel in the spatial domain. Specifically, the Epanechnikov kernel is employed.

## 4. Experimental results

In order to evaluate the performance of the proposed foveal attention system, the experiments have been divided into three parts: the comparison between uniform and foveal attention models; the evaluation of the ability of the approach for actively driving an image exploration process; and finally the evaluation of the attention and fixation prediction model. All tests have been conducted on an Intel(R) Core(TM)2 Duo CPU T8100 2.10 GHz.

### 4.1. Uniform vs. foveal attention

One of the main reasons for using a foveal strategy is the reduction of the computational costs. In our tests, running the system within different platforms, the foveal attention approach demonstrated to be approximately 4 times faster than its uniform counterpart. All tests were conducted using a Microsoft Kinect as input and working with images of 640 × 480 pixels. Within this framework, the algorithm is able to run at 10–12 frames per second (fps). The reduction on computational cost is significant, specially if we consider that the foveal image generation (the Kinect sensor provides an uniform image) is included in the computational costs associated to the foveal approach. If we remove these costs, the foveal approach is approximately 6 times faster.

**Figure 10:**
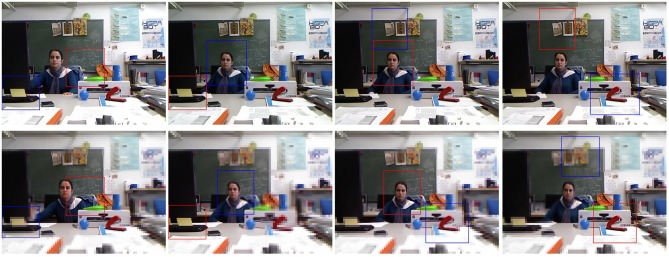
**Active exploration of a video sequence**. **(Top)** uniform images, and **(Bottom)** foveal images. In both cases the used color parameters have been τ_color_ = 50 and τ_percep_ = 100.

Then, the question is: what is the cost to pay for being faster? Figure 10 assesses the sequence of fixations obtained by an attention model that uses (top) uniform images and (bottom) foveal images. It must be noted that they are not the same video sequence, and although the scenario is the same for both trials (with the same relevant items), some differences can be presented due to light variations or slightly motions. In both cases the same set of weights has been employed for the saliency computation and the results are then very similar. There are significant differences on the peripheral part of the image, but the fovea is in both cases at the same resolution. And the fovea includes the object to attend.

**Figure 11:**
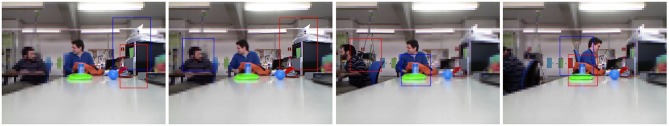
**Active exploration of a video sequence (see text for details) (τ_color_ = 50, τ_percep_ = 100)**.

On the contrary, the drawbacks of being slow are clear when dealing with real scenarios. Thus, Figure 11 shows how the use of foveal images is not sufficient to attend on time to a region marked as relevant (on the second frame of the sequence). When the fovea moves to this position (third frame), it does not find the searched region. The active exploration continues and the fovea will move to a new coherent position (the blue cup) on the next frame.

### 4.2. Active exploration using the foveal attention approach

**Figure 12:**
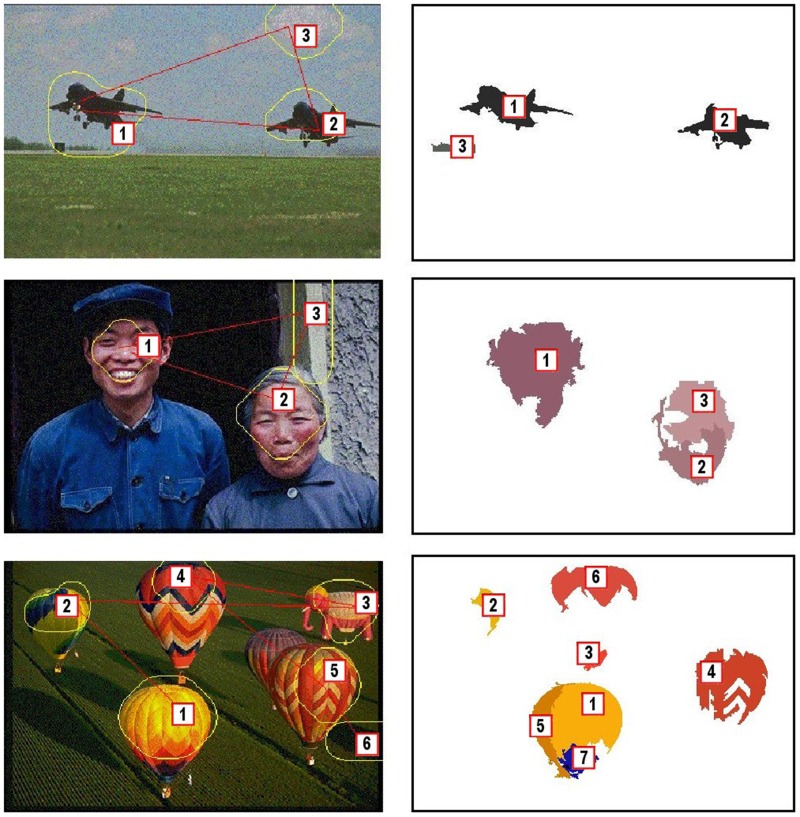
**Scanpath results for three images from the Saliency ToolBox**. **(Left)** Results obtained using the approach by Walther and Koch (2006), and **(Right)** sets of proto-objects obtained using our approach. Gaze ordering is drawn over the images.

**Figure 13:**
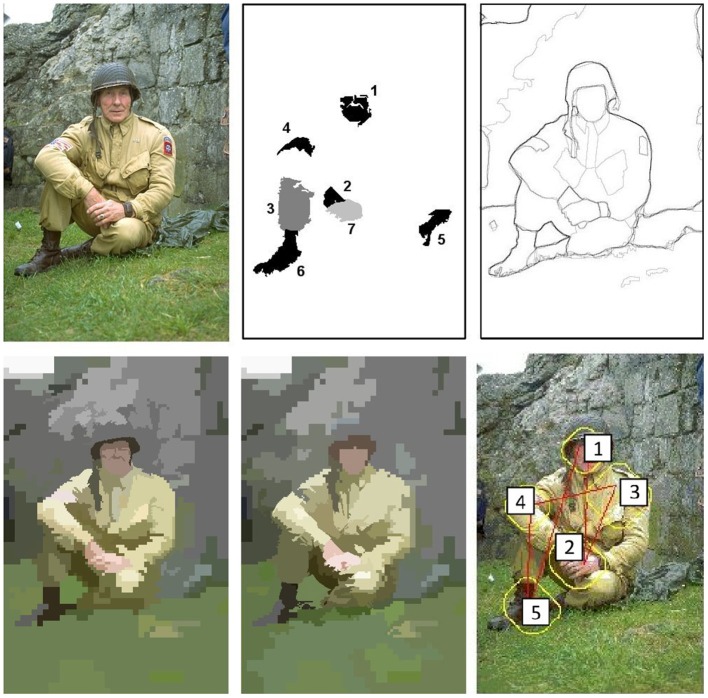
**Active exploration of the image #376043 of the Berkeley Segmentation Dataset**. **(Top left)** original image, **(Top middle)** set of seven first fixation regions, **(Top right)** human segmentations, **(Bottom left-middle)** first two segmentations from the proposed approach and **(Bottom right)** scanpath result using the approach by Walther and Koch (2006).

**Figure 14:**
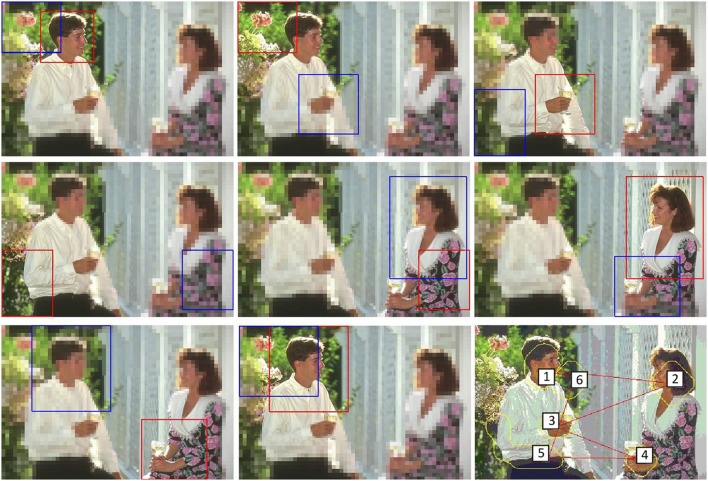
**Active exploration of the image #157055 of the Berkeley Segmentation Dataset**. From **(Top left)** to **(Bottom middle)**, the figure shows a sequence of fixations (each image shows the current fovea, marked with a red rectangle, and the next one, within a blue rectangle). **(Bottom right)** Scanpath result using the approach by Walther and Koch (2006).

As it has been illustrated in the previous section, due to its foveal nature, the proposed approach does not provide a single saliency map for a given scenario but a sequence of saliency maps. Thus, there is an iterative flow whose steps imply (a) to move the fovea to a new location, (b) to obtain a new saliency map, and (c) to determine the new location of the fovea according to this map. The foveal approach should then be understood within the framework of video processing, i.e., scenarios where visual information constantly changes due to ego-centric movements or dynamics of the world (Borji and Itti, 2013b). When we use this approach for exploring a static scene, the result will be the same: it is necessary more than one iteration to explore it (unless this has only one relevant object). Figure 12 shows scan-path results for three images from the Saliency ToolBox (http://www.saliencytoolbox.net/). The left column shows the results obtained using the approach by Walther and Koch (2006). The right one the set of proto-objects obtained using our approach. Gaze ordering is drawn over the images. Each iteration provides a foveal region to be analyzed in detail. This exploration is an active process which is completed in a finite number of iterations (when all the relevant parts of the image have been located at the fovea). This behavior is due to the existence of an IOR mechanism but also to the existing differences among foveal segmentations results depending on the location of the fovea. The foveal region is segmented in detail while the level of detail decreases with the distance to the fovea. That is, the segmentation of the same region can be very different between iterations. This is illustrated in Figures 13, 14.

Figure 13 shows that the approach outcomes a fixation region in each iteration. This fixation region is the most salient proto-object inside the fovea. These proto-objects are usually among the set of segments in which the people divides up the image (face, one hand, one leg…). The top-middle image of the figure represent the first seven fixation regions. At the bottom (left and middle), the figure shows the first two segmentations. Although there is certain constancy on the boundaries, they are not identical. Segmentations will be more different when fixation regions are more distant on the image. For instance, this occurs in Figure 14. From top-left to bottom-middle, this figure shows a sequence of fixations. The current fovea is marked within a red rectangle and the next within a blue one. The first fovea is over the face of the man, then it moves to a salient flower on the top-left corner, then to the hand of the man… Sometimes, this scan-path does not follow the path we could desire: from the hand it now moves to the elbow of the man and, from here, to the dress of the woman. But we are dealing with an active process, and it will return to “relevant” (from our point-of-view) regions quickly. Finally, this image also shows how the IOR works. After some frames, the fovea returns to previously visited regions (the face of the man, his hand…). Results are similar to the ones provided by the approach by Walther and Koch (2006) (see the bottom-right images at Figures 13, 14).

The effectiveness of our approach has been verified with experiments performed on human eye gaze data. As ground truth scan-paths, we use the JUDD publicly available eye tracking dataset (Judd et al., 2009). This dataset records human gaze in a free viewing setting (1003 images with scan-paths of 15 subjects). Our estimated scan-paths are obtained as an ordered sequence of region's centroids. The comparison between an estimated scan-path and one of these ground truth scan-paths is performed using the similarity index described by Liu et al. (2013). In this measuring metric, there is a parameter (*gap*), which is the penalty value employed when it is necessary to add a gap (deletion or insertion operation) in any of the scan-paths during local alignment. It is set to -1/2 in our tests. Finally, for each image at the JUDD dataset, we have 15 ground truth scan-paths (one from each users). Then, we compare each scan-path with all these ground truth ones, providing the average similarity value. Our result is close to 1.05. It can be noted that our approach provides a better result in this framework than the approaches by Itti et al. (1998) and Walther and Koch (2006) (both under 0.9). On the other hand, this result is under the Liu et al. (2013)'s scores (close to 1.15). However, it should be appreciated that the Liu et al. (2013)'s approach does not only use low-level feature saliency, but also spatial position and semantic content. Our approach does not take into account these factors.

### 4.3. Experiments with attention and fixation prediction

**Figure 15:**
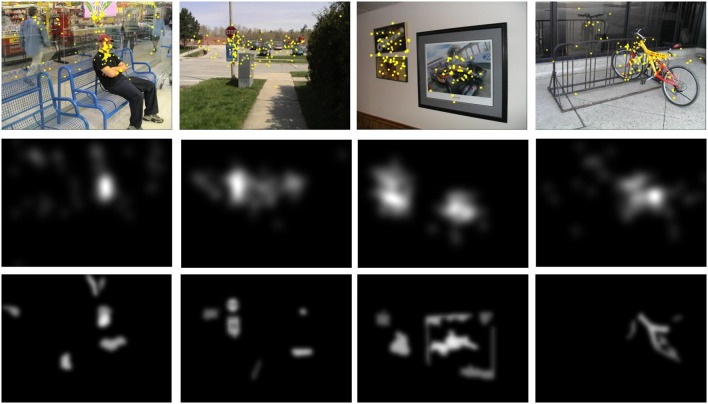
**Toronto Database**. **(Top)** original images and fixation points, **(Middle)** fixation density maps obtained from the human fixations, and **(Bottom)** fixation density maps obtained by the proposed foveal attention approach.

The approach has been evaluated using the Toronto database (Bruce and Tsotsos, 2009). This dataset was recently defined as the most widely used image data set in the review paper by Borji and Itti (2013b). The dataset contains 120 images (681 × 511 px) with eye-tracking data from 20 people. The subjects saw the images for four seconds, and they had no assigned task (i.e., free-viewing). Figure 15 shows four images of the data set. Fixations are drawn over the images. A fixation density map is generated for each image based on these fixation points (Bruce and Tsotsos, 2009). They are also shown at Figure 15 under each original image.

Contrary to the most attention approaches, our saliency maps should be also estimated from a set of fixations. However, contrary to the density maps obtained from experimental human eye tracking data, our fixations cannot be associated to points, but to regions. The fixation density maps shown at the bottom row of the Figure 15 were built by the sum of the most saliency regions on *n* fixations. The number *n* was equal to the mean of the number of human fixations recorded for this image in the original data set.

Then, we use the well-known receiver operating characteristic (ROC) area under curve (AUC) measure to assess the performance of the approach. Each saliency map can be thresholded and then considered to be a binary classifier that separates positive samples (fixation points of all subjects on that image) from negative samples (fixation points of all subjects on all other images in the database). This process avoids the center-bias effect (Borji and Itti, 2013b). Then, we can sweep over all thresholds to estimate the ROC curve for each saliency map and calculated the area beneath the ROC curve. This area provides a good measure to assess how accurately the saliency map predicts the eye fixations on the image. An AUC value greater than 0.5 indicates positive correlation. As a performance baseline we can estimate an ideal AUC measuring how well the fixations of one subject can be predicted by the fixations of the rest of subjects. The ideal AUC for the data set is 0.878 (Borji and Itti, 2013b). In our experiments, the obtained score was 0.669. This value is similar to the ones provided by other methods. In the ranking documented by Borji and Itti (2013a), it will be the fifth best value of 28 evaluated models.

## 5. Conclusions and future work

We proposed in this paper a foveal model of attention which combines static cues with depth and tracking to deal with dynamic scenarios. The framework was developed for an active observer, but this paper shows that it can also be applied to image databases. These static images were preferably employed to compare or evaluate the approach. Contrary to other approaches (such as the recently proposed by Mishra et al., 2012), we do not pursuit here a novel formulation of segmentation. Thus, in Section 4.2, we prefer to speak about active exploration and not segmentation. Active segmentation will probably require an additional (and better) algorithm that will try to extract the whole object from the fixation region. We refer the reader to the excellent work by Mishra et al. (2012) to understand the whole problem of active segmentation.

With respect to previous approaches to object-based attention, this work must be classified with those methods that compute the saliency of scene regions and not of isolated pixels. For this end, these approaches segment the input image before to evaluate and obtain the saliency map. As a main difference with previous works such as the ones by Orabona et al. (2007) and Yu et al. (2010), our approach performs this segmentation as a multi-resolution process, where only the fovea is processed with details. Thus, this segmentation depends on the position of the last fovea or ROI. Furthermore, our framework provides a complete approximation for closing the loop that involves segmentation and saliency estimation, including an inhibition of return mechanism. We consider that analyzing this loop closing is basic to understand an object-based attention mechanism working on a real, dynamic scenario.

This approach should be extended in several ways. Launched as a system to endow into a mobile robot, the foveal approach needs to be faster and to take into consideration top-down factors. We are working on both research direction. The speed will be improved by implementing the approach in a Zedboard platform. This is allowing to move part of the code to a FPGA, meanwhile the main function continues running on a processor. Top-down component of attention will initially come from the adjustment of the weights used to bias the saliency maps. Further work should be addressed to add object models on this process.

### Conflict of interest statement

The authors declare that the research was conducted in the absence of any commercial or financial relationships that could be construed as a potential conflict of interest.
